# Comparable Acute Antidepressant-like Effects of Two Subanesthetic Ketamine Doses Are Associated with Divergent Region-Specific Molecular Responses After Adolescent Chronic Stress

**DOI:** 10.3390/ijms27209126

**Published:** 2026-10-15

**Authors:** Ana Zivanovic, Iva Lukic, Emilija Glavonic, Zeljka Brkic, Milos Mitic, Sanja Ivkovic, Miroslav Adzic

**Affiliations:** 1 Vinca—Institute for Nuclear Sciences, National Institute of Republic of Serbia, University of Belgrade, Mike Petrovica Alasa 12–14, Vinca, 11000 Belgrade, Serbia; anazivanovicmb@gmail.com (A.Z.); iva.lukic@vinca.rs (I.L.); emilija.glavonic@vin.bg.ac.rs (E.G.); milos.mitic@vin.bg.ac.rs (M.M.); miraz@vin.bg.ac.rs (M.A.); 2 Department of Biomedicine, Aarhus University, Høegh-Guldbergs Gade 10, Building 1242, room 443, 8000 Aarhus, Denmark; zeljka.b@biomed.au.dk

**Keywords:** chronic unpredictable stress (CUS), depression, ketamine, subanesthetic dose

## Abstract

Ketamine produces rapid antidepressant-like effects across a range of subanesthetic doses, yet how dose shapes behavioral efficacy and region-specific molecular signaling following adolescent chronic unpredictable stress (CUS) is not well understood. Here, we examined the behavioral and molecular consequences of two subanesthetic ketamine doses in adult mice with depression-like behavior induced by CUS. Male C57BL/6J mice were exposed to CUS during adolescence and developed depression-like behavior in adulthood. Adult mice received a single injection of vehicle or ketamine at 6 or 10 mg/kg, while non-stressed controls received vehicle. Both ketamine doses reduced immobility in the tail suspension test 24 h after treatment, indicating comparable acute antidepressant-like effects; however, this effect was not sustained one week later. Molecular analyses of the prefrontal cortex, hippocampus, and striatum were performed 24 h after behavioral testing, corresponding to 48 h after ketamine administration, to define region-specific ERK, mTOR, JNK, and BDNF signaling profiles. These analyses revealed dose- and region-dependent regulation of all four markers. The higher ketamine dose produced more extensive molecular changes without producing a greater reduction in tail-suspension test (TST) immobility, suggesting that the behavioral relevance of ketamine-induced signaling depends on regional context rather than the overall magnitude of pathway activation.

## 1. Introduction

Major depressive disorder (MDD) remains insufficiently treated in a substantial proportion of patients. Conventional antidepressants usually require weeks to produce clinical improvement and often fail to provide adequate symptom relief [1,2,3]. Ketamine, a noncompetitive N-methyl-D-aspartate (NMDA) receptor antagonist, has therefore attracted considerable attention because subanesthetic doses can induce rapid antidepressant effects, often within hours after a single administration [4,5].

Ketamine’s rapid antidepressant-like effects are primarily associated with glutamate-dependent activation of plasticity-related signaling. BDNF is considered a central mediator of this response, acting through downstream pathways that include ERK and mTOR, which regulate synaptogenesis, dendritic remodeling, and functional connectivity [6]. However, ketamine-induced molecular responses are not limited to these canonical plasticity pathways. Recent work in adult mice with depression-like behavior induced by adolescent chronic unpredictable stress (CUS) showed that ketamine also affects stress-sensitive kinase signaling, including JNK, in a region-dependent manner [7]. This supports the analysis of BDNF-, ERK-, mTOR-, and JNK-related signaling as complementary molecular readouts of ketamine response after adolescent stress.

Chronic stress is a major precipitating factor in depression [8,9]. Stress exposure during adolescence is especially important because this developmental period is associated with ongoing maturation of corticolimbic and reward-related circuits. Accordingly, adolescent CUS can produce persistent behavioral and molecular alterations resulting in depression-like behavior that extends into adulthood [7,10,11,12]. These long-term effects are unlikely to be uniform across the brain, as regions involved in affective regulation, stress adaptation, memory, motivation, and reward differ in their vulnerability to stress and their contribution to antidepressant responses.

The prefrontal cortex, hippocampus, and striatum represent functionally distinct but interconnected components of these circuits. The prefrontal cortex contributes to affective regulation, cognitive control, and adaptive responses to stress and is consistently implicated in major depressive disorder [13,14]. The hippocampus is highly sensitive to chronic stress and participates in contextual processing, memory, emotional regulation, and antidepressant-related neuroplasticity [15,16]. The striatum, particularly its ventral component, is central to motivation and reward processing, and altered striatal function has been associated with anhedonia and reduced reward responsiveness in depression [16,17]. Parallel analysis of these regions can therefore reveal whether ketamine produces a common molecular response across stress-related circuits or engages region-specific signaling programs.

Dose selection remains an important issue in ketamine research, as antidepressant-like efficacy must be balanced against dose-related adverse effects, including psychotomimetic, dissociative, locomotor, and abuse-related effects [18]. A lower subanesthetic dose that preserves behavioral efficacy could therefore be advantageous, particularly if it achieves comparable behavioral effects without recruiting additional molecular responses. However, most preclinical studies have examined ketamine at a single effective dose or have compared doses mainly at the behavioral level. Available dose-comparison studies indicate that increasing the ketamine dose does not necessarily enhance antidepressant-like efficacy and may instead produce different molecular and synaptic responses [4]. Importantly, much of the available dose-comparison evidence is derived from comparisons between an antidepressant-effective low dose and substantially higher doses commonly used in psychotomimetic or schizophrenia-related paradigms. Whether more closely spaced doses within the antidepressant-effective subanesthetic range produce equivalent or distinct molecular responses remains considerably less clear. This distinction is important because additional molecular pathway engagement at a higher dose may not necessarily translate into greater behavioral efficacy. Defining these differences may help identify signaling changes associated with behavioral improvement and distinguish them from additional molecular responses recruited at higher doses.

The present study investigated whether ketamine at 6 and 10 mg/kg differentially regulates BDNF-, ERK-, mTOR-, and JNK-related signaling in the prefrontal cortex, hippocampus, and striatum of mice exposed to adolescent CUS. These doses were selected to compare two effective subanesthetic levels within the range commonly used in rodent models of antidepressant-like activity, where doses up to 10 mg/kg frequently produce behavioral effects [4,19,20]. Molecular analyses were performed 48 h after ketamine administration. By integrating behavioral assessment with region-specific molecular analyses, we aimed to determine whether comparable behavioral responses to the two ketamine doses are accompanied by convergent molecular signaling or, alternatively, by dose- and region-specific patterns of intracellular pathway activation.

## 2. Results

### 2.1. Both Ketamine Doses Produce Comparable Acute but Transient Antidepressant-like Effects Following CUS

**Figure 1:**
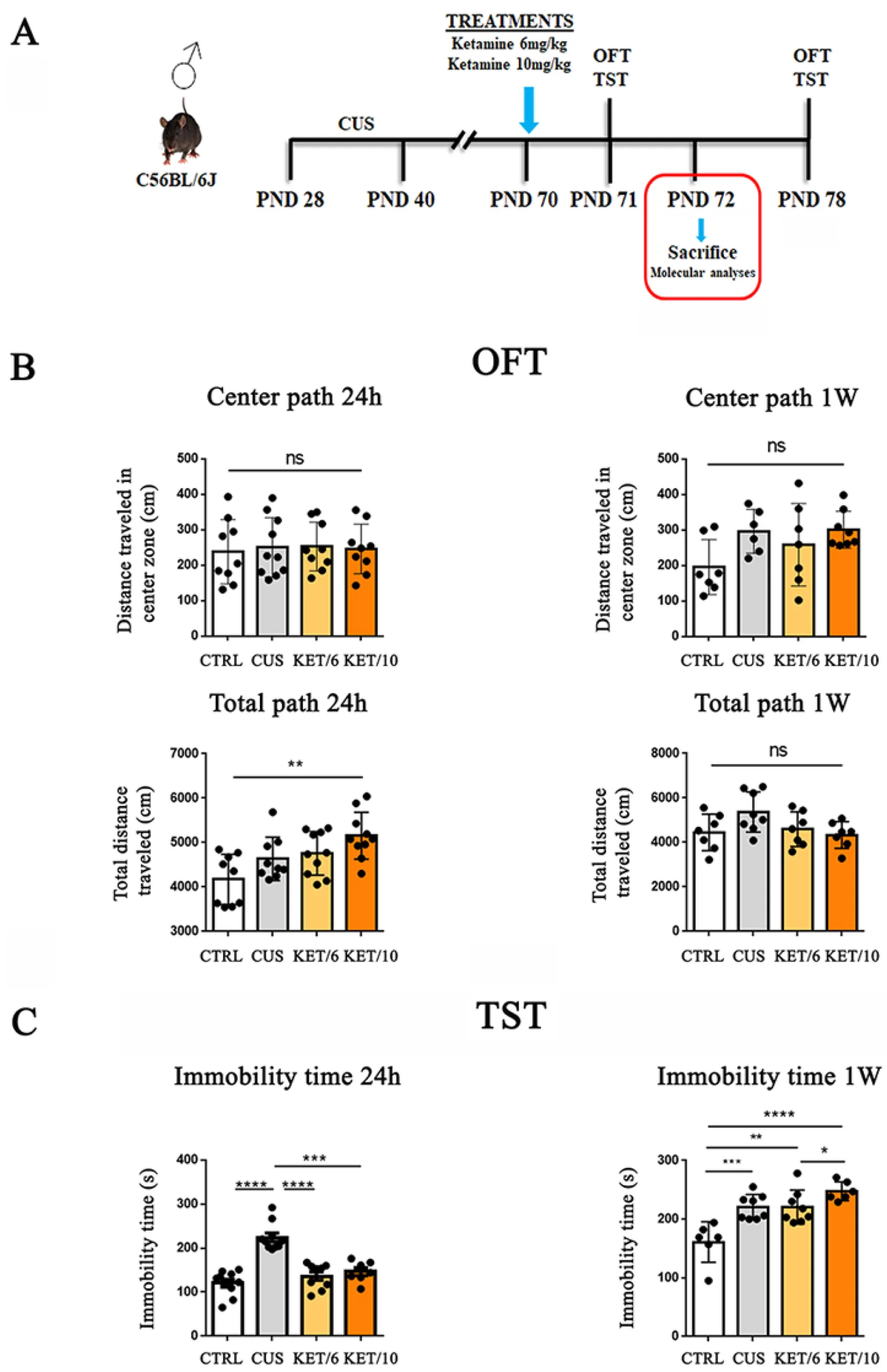
The effects of two subanesthetic ketamine doses on locomotor activity and TST immobility. The 24-h and one-week behavioral outcomes presented in this study were obtained from two independent experimental cohorts. (**A**) Experimental design showing the timeline of behavioral testing (OFT and TST) and molecular analyses. (**B**) Distance traveled in the center zone (center path) in the OFT measured 24 h after treatment (top left); center path in the OFT measured one week after treatment (top right); total distance traveled (total path) in the OFT measured 24 h after treatment (bottom left) and total path in the OFT measured one week after treatment (bottom right). (**C**) Immobility time in the TST assessed 24 h post-treatment (left); Immobility time in the TST assessed one week post-treatment (right). Data are expressed as mean ± SEM, with differences among all groups assessed by one-way ANOVA followed by post hoc comparisons. N = 6–10 animals per group. * *p* < 0.05, ** *p* < 0.01, *** *p* < 0.001, **** *p* < 0.0001. Abbreviations: OFT, open field test; TST, tail suspension test; CTRL, control group; CUS, chronic unpredictable stress; ns, not significant; KET/6, ketamine 6 mg/kg; KET/10, ketamine 10 mg/kg.

To evaluate the effects of CUS and subanesthetic ketamine treatment on locomotor activity and depression-like behavior, mice were tested 24 h and one week after treatment using the open field test (OFT) [21] and the tail suspension test (TST) [22]. The experimental timeline is shown in Figure 1A.

In the OFT, no significant differences in center-zone distance were detected among groups at either 24 h or one week. Total distance traveled at 24 h was significantly increased in the KET/10 group compared with CTRL, whereas no significant differences were observed among the remaining groups. At one week, total distance traveled did not differ among groups (Figure 1B).

In the TST, CUS markedly increased immobility at 24 h, by approximately 79% relative to CTRL. Both ketamine doses reduced immobility by approximately 40% compared with CUS, with values approaching those of non-stressed controls. No significant difference was detected between KET/6 and KET/10, indicating comparable acute antidepressant-like effects (Figure 1C).

At one week, immobility remained significantly elevated relative to CTRL in the CUS, KET/6, and KET/10 groups, indicating that the acute reduction in immobility was not sustained. Moreover, immobility in the KET/10 group was significantly higher than in the KET/6 group, by approximately 12%. Thus, although both doses produced comparable antidepressant-like effects at 24 h, this effect was transient, and the higher dose was associated with greater immobility at the later time point (Figure 1C).

### 2.2. Different Subanesthetic Ketamine Doses Produce Distinct Molecular Responses Across Brain Regions

Phosphorylated and total ERK, mTOR, and JNK protein levels were examined in the prefrontal cortex, hippocampus, and striatum 48 h after ketamine administration, corresponding to 24 h after BTs. Phospho-to-total protein ratios were calculated as indices of relative pathway activation. These pathways were selected as complementary readouts of stress- and plasticity-related signaling, allowing the behavioral effects observed in the OFT and TST to be interpreted in the context of underlying molecular adaptations.

#### 2.2.1. Prefrontal Cortex

**Figure 2:**
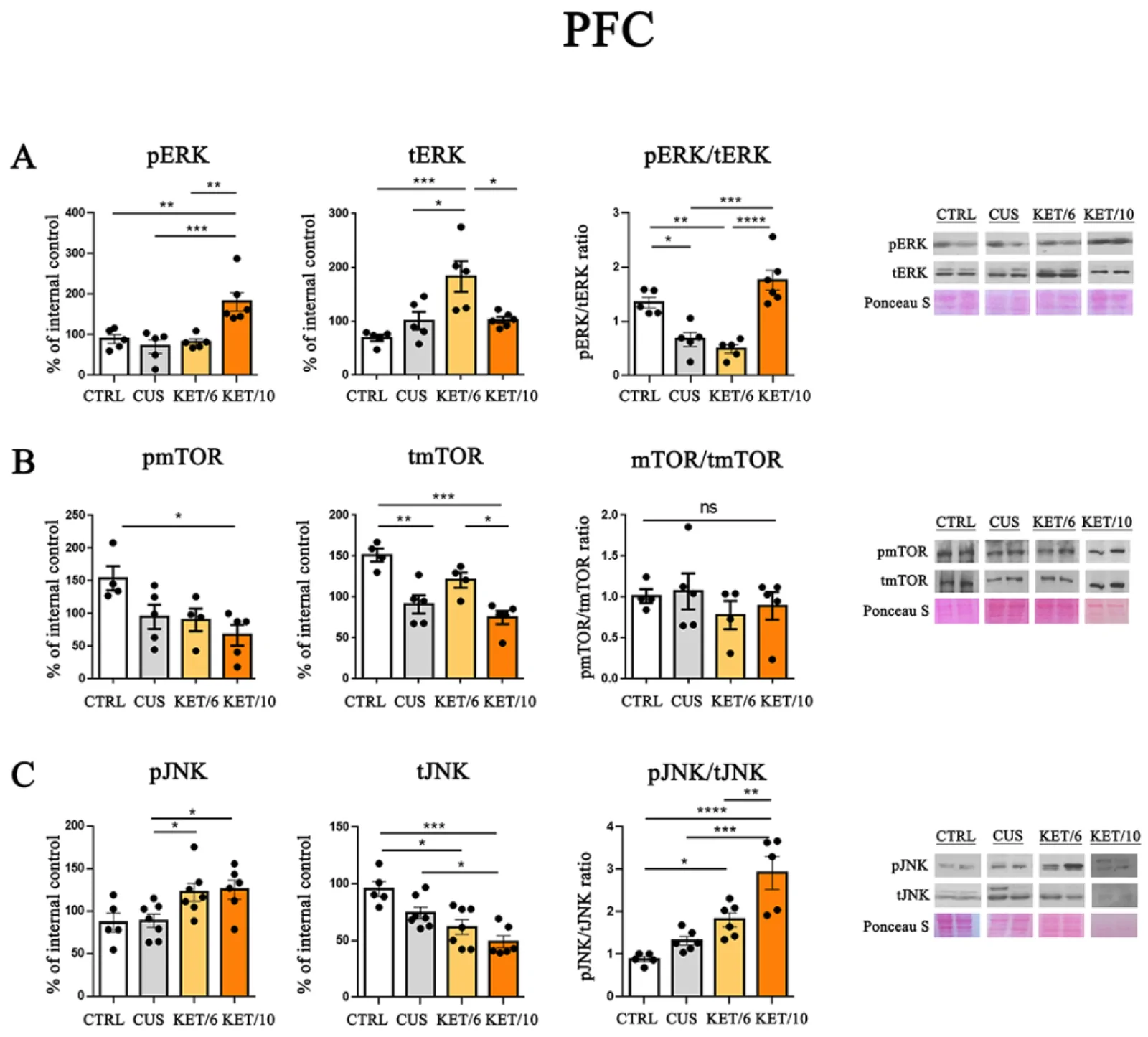
The effects of two subanesthetic ketamine doses on ERK, mTOR, and JNK signaling in the PFC. (**A**) Phosphorylated (pERK), total (tERK), and phospho-to-total ERK ratio. (**B**) Phosphorylated (pmTOR), total (tmTOR), and phospho-to-total mTOR ratio. (**C**) Phosphorylated (pJNK), total (tJNK), and phospho-to-total JNK ratio. N = 4–7 animals per group. Data are expressed as mean ± SEM. Differences among groups were assessed by one-way ANOVA followed by Tukey’s multiple comparisons test; ns—not significant. * *p* < 0.05, ** *p* < 0.01, *** *p* < 0.001, **** *p* < 0.0001.

In the PFC, CUS and ketamine treatment produced pathway-specific changes in ERK, mTOR, and JNK activation. CUS reduced the pERK/ERK ratio by 54.1% relative to CTRL, and KET/6 did not reverse this reduction, with the ratio remaining 63.7% below control levels. In contrast, KET/10 increased the pERK/ERK ratio by 183.9% relative to CUS and by 259.2% relative to KET/6, restoring relative ERK activation to control levels (Figure 2A). mTOR activation was not significantly altered, as the pmTOR/mTOR ratio remained unchanged among groups despite changes in phosphorylated and total mTOR protein levels (Figure 2B). JNK activation in the PFC showed a dose-dependent pattern. CUS did not significantly alter the p-JNK/JNK ratio relative to CTRL, whereas KET/6 and KET/10 significantly increased JNK activation relative to CTRL, by 103.4% and 227.0%, respectively. KET/10 also significantly increased the p-JNK/JNK ratio by 120.5% relative to CUS and by 60.8% relative to KET/6, whereas KET/6 did not significantly differ from CUS (Figure 2C).

#### 2.2.2. Hippocampus

**Figure 3:**
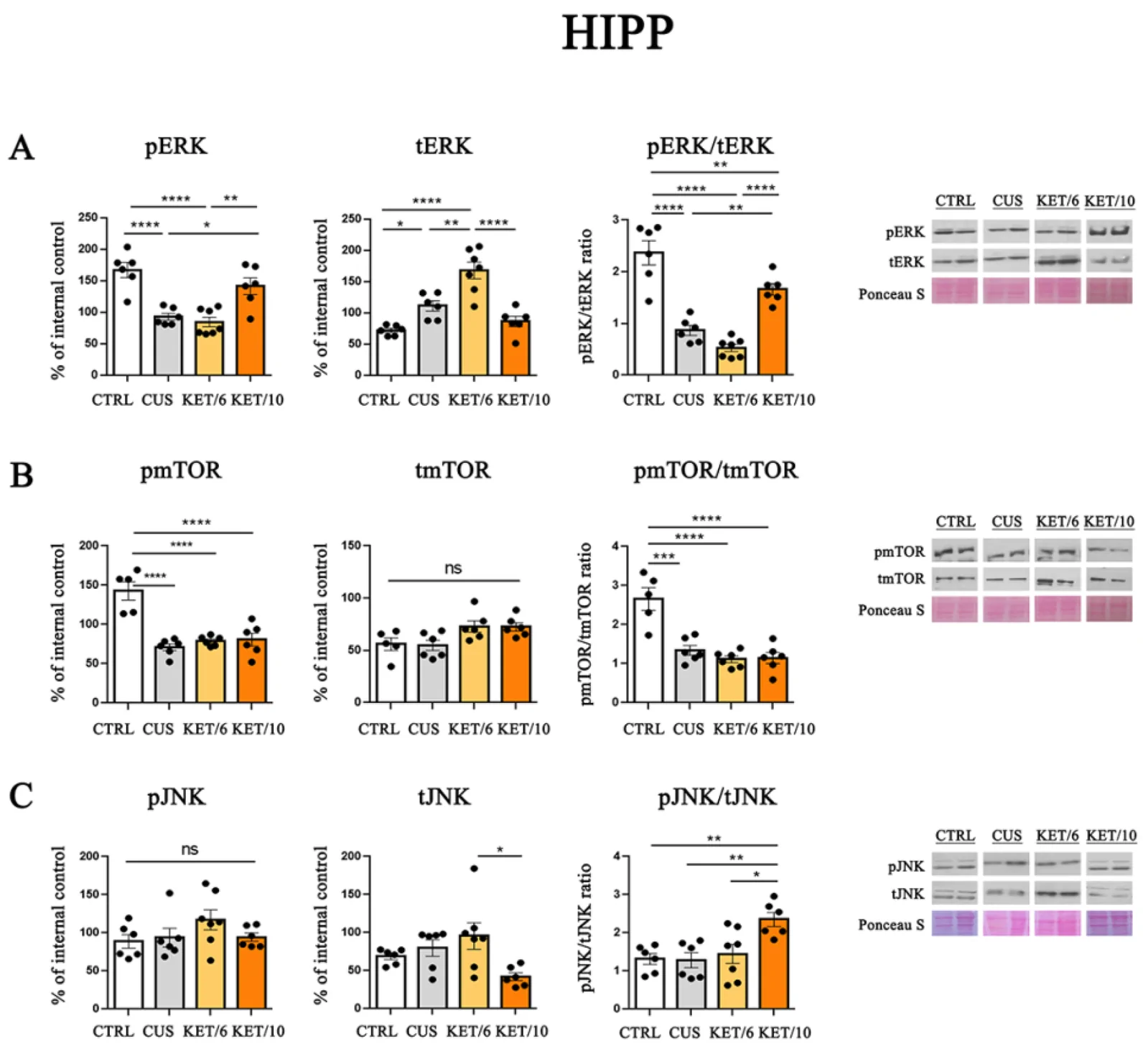
The effects of two subanesthetic ketamine doses on ERK, mTOR, and JNK signaling in the hippocampus. (**A**) Phosphorylated (pERK), total (tERK), and phospho-to-total ERK ratio. (**B**) Phosphorylated (pmTOR), total (tmTOR), and phospho-to-total mTOR ratio. (**C**) Phosphorylated (pJNK), total (tJNK), and phospho-to-total JNK ratio. N = 5–7 animals per group. Data are expressed as mean ± SEM. Differences among groups were assessed by one-way ANOVA followed by Tukey’s multiple comparisons test; ns—not significant, * *p* < 0.05, ** *p* < 0.01, *** *p* < 0.001, **** *p* < 0.0001.

In the hippocampus, CUS and ketamine treatment produced pathway-specific changes in ERK, mTOR, and JNK activation, with the clearest dose-dependent differences observed for ERK and JNK. CUS reduced the p-ERK/ERK ratio by 63.1% relative to CTRL, while KET/6 further reduced the ratio to 85.2% below CTRL levels, although it did not significantly differ from CUS. In contrast, KET/10 increased the ratio by 90.8% relative to CUS and by 374.3% relative to KET/6, although it remained 29.7% below CTRL levels, bringing relative ERK activation closer to control levels (Figure 3A). Hippocampal mTOR showed a uniform reduction across all CUS-exposed groups. The pmTOR/mTOR ratio was decreased by approximately 55% in CUS, KET/6, and KET/10 relative to CTRL, indicating that neither ketamine dose restored relative mTOR activation (Figure 3B). JNK showed a different dose-related pattern. CUS did not significantly alter the p-JNK/JNK ratio relative to CTRL, whereas KET/10 increased the p-JNK/JNK ratio by approximately 124% compared with the other groups, while KET/6 did not significantly affect this ratio. Thus, increased relative JNK activation in the hippocampus was observed only after the higher ketamine dose (Figure 3C).

#### 2.2.3. Striatum

**Figure 4:**
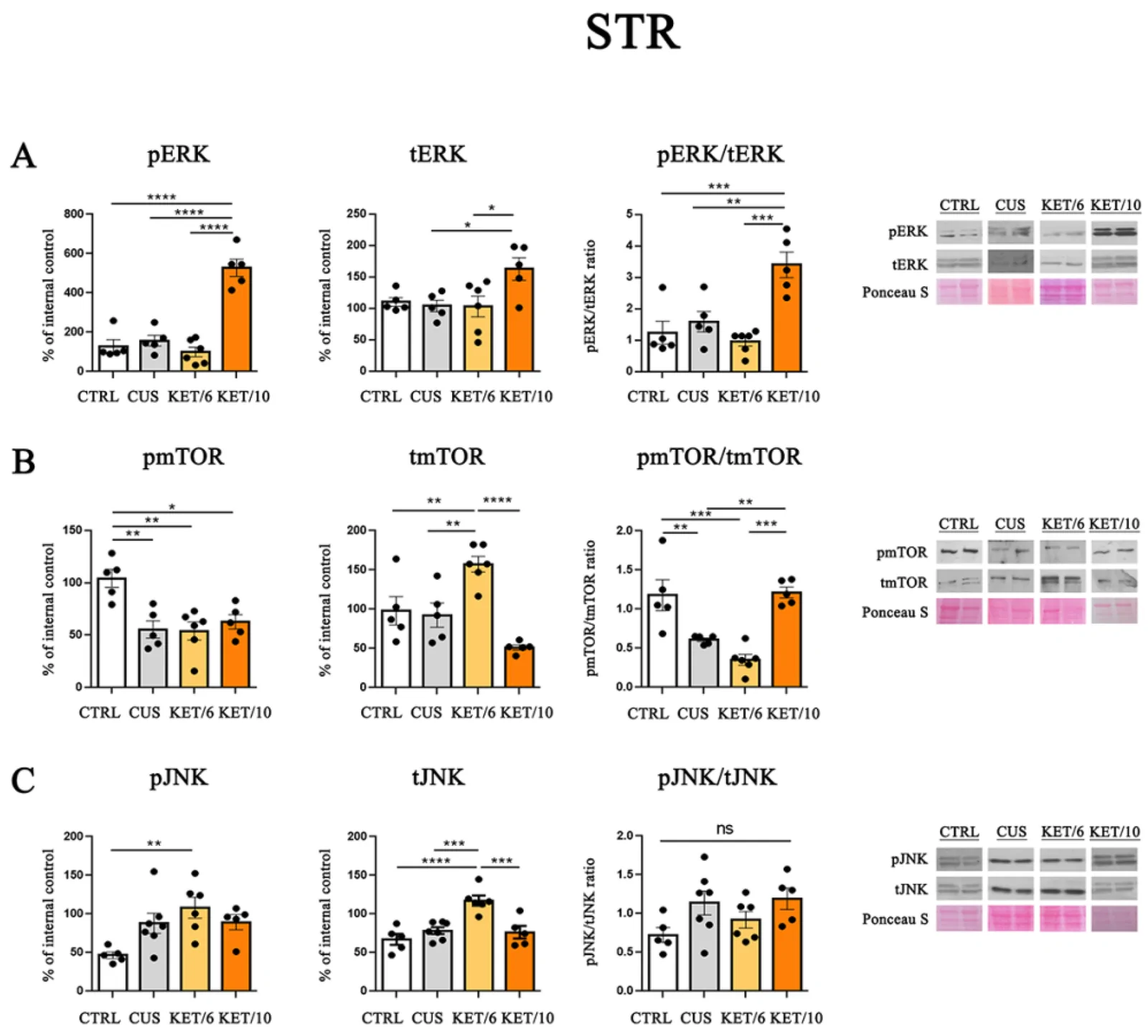
The effects of two subanesthetic ketamine doses on ERK, mTOR, and JNK signaling in the striatum. (**A**) Phosphorylated (pERK), total (tERK), and phospho-to-total ERK ratio. (**B**) Phosphorylated (pmTOR), total (tmTOR), and phospho-to-total mTOR ratio. (**C**) Phosphorylated (pJNK), total (tJNK), and phospho-to-total JNK ratio. N = 5–7 animals per group. Data are expressed as mean ± SEM. Differences among groups were assessed by one-way ANOVA followed by Tukey’s multiple comparisons test; ns—not significant, * *p* < 0.05, ** *p* < 0.01, *** *p* < 0.001, **** *p* < 0.0001.

In the striatum, ketamine produced a particularly pronounced dose-dependent modulation of ERK and mTOR signaling, whereas JNK activation was not altered. Striatal ERK activation differed clearly between the two ketamine doses. CUS did not significantly alter the p-ERK/ERK ratio relative to CTRL, whereas KET/10 increased the pERK/ERK ratio by 176.4% relative to CTRL, 113.8% relative to CUS, and 254.2% relative to KET/6, whereas KET/6 did not significantly differ from CUS. Thus, enhanced relative ERK activation in the striatum was observed only after the higher ketamine dose (Figure 4A). Striatal mTOR signaling also distinguished the two ketamine doses. CUS significantly reduced the p-mTOR/mTOR ratio by 47.9% relative to CTRL, while KET/6 further reduced the ratio to 70.1% below CTRL levels. In contrast, KET/10 significantly increased the p-mTOR/mTOR ratio by 98.4% relative to CUS and by 245.7% relative to KET/6, although it did not significantly differ from CTRL. Thus, KET/10 restored relative mTOR activation toward control levels, whereas KET/6 did not (Figure 4B). By contrast, the p-JNK/JNK ratio did not differ significantly between CTRL and CUS or among the ketamine-treated groups, indicating that relative JNK activation in the striatum was not affected by CUS or ketamine treatment (Figure 4C).

#### 2.2.4. Ketamine Regulates BDNF Expression in a Region- and Dose-Dependent Manner

**Figure 5:**
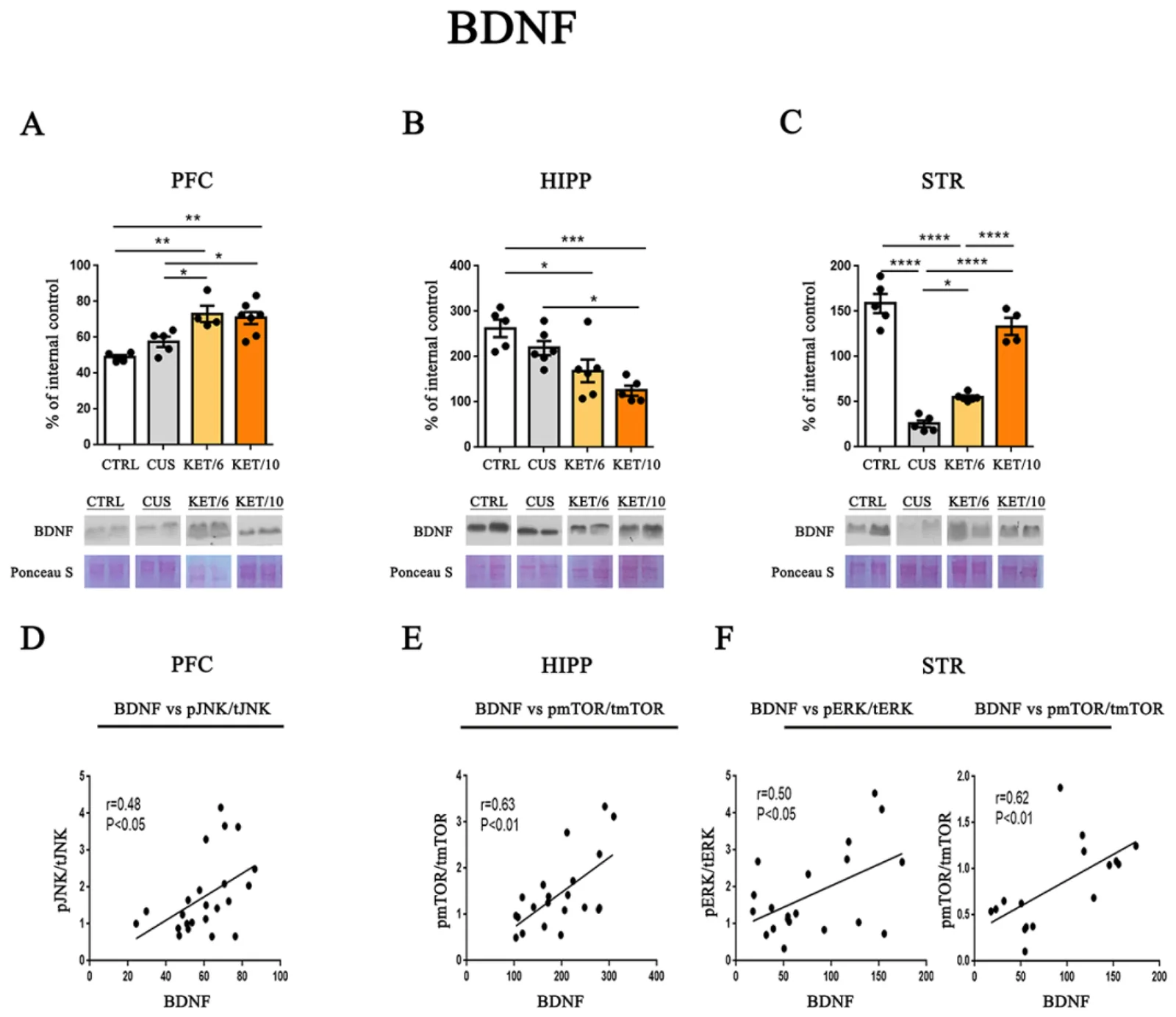
The effects of two subanesthetic ketamine doses on BDNF expression and BDNF–kinase correlations across brain regions. BDNF expression in the PFC (**A**), hippocampus (**B**), and striatum (**C**). Differences among groups were assessed by one-way ANOVA followed by Tukey’s multiple comparisons test. N = 4–7 animals per group. * *p* < 0.05, ** *p* < 0.01, *** *p* < 0.001, **** *p* < 0.0001. Pearson correlations between BDNF expression and proteins in the PFC (**D**), HIPP (**E**) and STR (**F**). All animals combined. Data are expressed as mean ± SEM.

BDNF expression showed region-dependent responses to ketamine treatment. In the PFC, CUS did not significantly alter BDNF levels, whereas both ketamine doses increased expression by approximately 46% relative to CTRL and by approximately 24% relative to CUS, with no difference between KET/6 and KET/10 (Figure 5A). In the hippocampus, BDNF was also unchanged by CUS alone, but ketamine treatment reduced its expression. KET/6 decreased BDNF by 35.6% relative to CTRL, while KET/10 produced a larger numerical reduction of 52.4% relative to CTRL and 42.9% relative to CUS (Figure 5B). The striatum showed a different pattern, dominated by a marked CUS-induced reduction and dose-dependent recovery after ketamine. CUS reduced BDNF by 83.6% relative to CTRL, while KET/6 decreased BDNF by 65.3% relative to CTRL but increased it by 111.5% relative to CUS. KET/10 produced a 411.5% increase relative to CUS and reached levels 141.8% higher than those observed after KET/6, approaching CTRL values (Figure 5C). Thus, ketamine increased BDNF in the PFC, decreased it in the hippocampus, and restored stress-reduced BDNF in the striatum, with the clearest dose-dependent effect observed in the striatum.

Correlation analyses further showed that BDNF was associated with different kinase pathways across regions. In the PFC, BDNF correlated positively with the pJNK/JNK ratio (r = 0.48; *p* < 0.05). In the hippocampus, it correlated with the pmTOR/mTOR ratio (r = 0.63; *p* < 0.01), whereas in the striatum it correlated with both pERK/ERK and pmTOR/mTOR ratios (r = 0.50; *p* < 0.05 and r = 0.62; *p* < 0.01, respectively; Figure 5D–F).

#### 2.2.5. Associations Between TST Immobility and Regional Molecular Signaling

To examine associations between TST immobility and regional molecular signaling, exploratory Pearson correlations were performed between TST immobility and ERK, mTOR, JNK, and BDNF measurements obtained from corresponding animals in the PFC, hippocampus, and striatum. Correlations were first examined across the complete experimental population (CTRL, CUS, KET/6, and KET/10), thereby assessing molecular measures associated with the overall behavioral phenotype across stress and treatment conditions. In addition, correlations were analyzed separately within the KET/6 and KET/10 groups to determine whether associations were also detectable among animals receiving the same ketamine dose. Correlations based on fewer than five paired observations were not interpreted.

**Figure 6:**
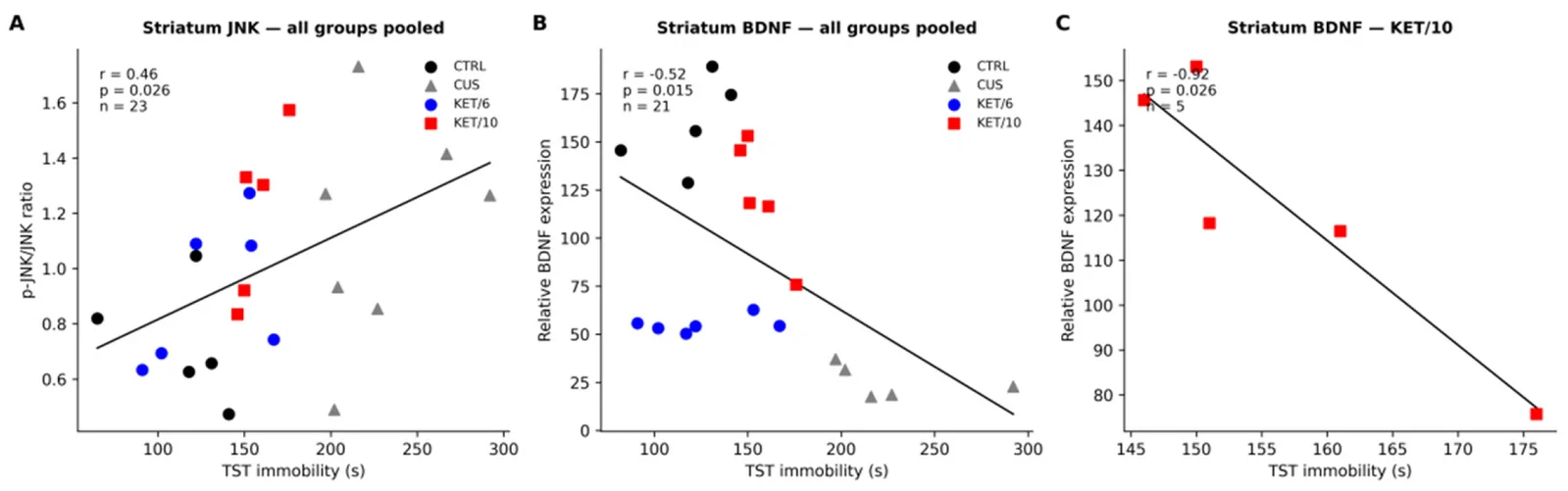
Associations between TST immobility and molecular markers. Pearson correlations were calculated between individual TST immobility values and molecular measurements obtained from corresponding animals. (**A**) Striatal p-JNK/JNK ratio across the complete experimental population (CTRL, CUS, KET/6, and KET/10). (**B**) Striatal BDNF expression across the complete experimental population. (**C**) Striatal BDNF expression within the KET/10 group. Pearson r, *p*-value, and n are indicated in each panel. Correlation analyses were considered exploratory.

Across all experimental groups, TST immobility was positively correlated with striatal p-JNK/JNK (r = 0.46, *p* = 0.026, n = 23; Figure 6A), indicating that higher striatal JNK activation was associated with greater immobility. In contrast, striatal BDNF expression was negatively correlated with TST immobility (r = −0.52, *p* = 0.015, n = 21; Figure 6B), with higher BDNF levels associated with lower immobility. No significant correlations were detected between TST immobility and ERK, mTOR, or the remaining BDNF and JNK measures in the PFC or hippocampus.

Dose-specific analyses revealed no significant correlations within the KET/6 group. Within the KET/10 group, however, striatal BDNF expression showed a strong negative association with TST immobility (r = −0.92, *p* = 0.026, n = 5; Figure 6C). Thus, animals receiving KET/10 with higher striatal BDNF levels showed lower TST immobility. The complete correlation matrices for the all-group and dose-specific analyses are presented in Supplementary Figures S1 and S2, respectively.

## 3. Discussion

The present study shows that two subanesthetic ketamine doses can produce comparable acute antidepressant-like effects while engaging markedly different molecular responses across brain regions. Both 6 and 10 mg/kg ketamine reduced immobility 24 h after treatment in mice exposed to adolescent CUS, indicating that the lower dose was sufficient to produce the comparable acute behavioral response observed in this model. However, this behavioral similarity was not accompanied by molecular equivalence. The higher dose produced broader activation of ERK- and mTOR-related signaling, together with stronger restoration of striatal BDNF, whereas the molecular response to 6 mg/kg was more restricted. Thus, the main finding of this study is not simply that ketamine regulates intracellular signaling in a dose-dependent manner, but that greater molecular engagement does not necessarily translate into greater antidepressant-like efficacy.

The increase in total OFT distance observed after 10 mg/kg ketamine should also be considered when interpreting the acute TST response, because increased locomotor activity can potentially influence immobility-based measures. However, KET/10 did not significantly differ from the CUS group in total distance traveled, whereas both ketamine doses significantly reduced TST immobility relative to CUS. Moreover, KET/6 produced a comparable reduction in immobility without detectable locomotor stimulation. These findings support an antidepressant-like effect of both ketamine doses while further indicating that the lower dose achieved a comparable behavioral response without the additional locomotor alteration observed after the higher dose.

ERK, mTOR, JNK, and BDNF are widely implicated in ketamine-induced plasticity and stress-related signaling [6,23,24,25,26]. However, their functional significance is strongly influenced by brain region and temporal context. The present data therefore provide an opportunity to examine how two behaviorally effective ketamine doses differentially engage these pathways across the PFC, hippocampus, and striatum.

The principal dose- and region-dependent molecular responses are summarized in Table 1.

The ERK findings illustrate the dissociation between molecular engagement and behavioral efficacy. In the PFC and hippocampus, ketamine dose of 10 mg/kg restored relative ERK activation that was reduced after CUS, whereas in the striatum it induced a pronounced increase above control levels. However, this stronger ERK response did not produce greater acute behavioral efficacy than 6 mg/kg, consistent with evidence that enhanced ERK signaling can augment the persistence of ketamine’s antidepressant-like effects without necessarily increasing the magnitude of the acute behavioral response [27]. This finding should be considered in the context of the strongly time-dependent nature of ketamine-induced ERK signaling. Ma et al. [28] reported increased ERK phosphorylation 6 h after ketamine administration and showed that this activation was transient in mature hippocampal neurons, with p-ERK no longer detected in the granular layer at 24 h. Importantly, pharmacological inhibition of MEK–ERK signaling before ketamine administration blocked the antidepressant-like behavioral response assessed 24 h later, supporting a role for ERK signaling in the development of ketamine-induced adaptations. In the present study, ERK signaling was assessed 48 h after ketamine administration, and therefore the observed region-specific changes are more likely to reflect later molecular adaptations than the initial signaling events triggered by ketamine. Similarly, in our previous adolescent CUS study, sustained antidepressant-like effects of the combined ketamine-NorBNI treatment at one week were accompanied by increased ERK activation in the PFC and striatum [7]. Thus, the ERK changes observed here may reflect later plasticity-related adaptations rather than signaling directly related to the magnitude of the acute behavioral response.

The pronounced striatal ERK response after 10 mg/kg was accompanied by increased mTOR activation and recovery of stress-reduced BDNF levels, suggesting coordinated recruitment of plasticity-related signaling in this region. This is notable because the striatum contributes to reward processing, motivation, action selection, and anhedonia-related behavior, and altered striatal reward responsiveness has been repeatedly implicated in major depressive disorder [16,17,29,30,31]. The coordinated regulation of these pathways may therefore reflect a regionally integrated plasticity response to the higher ketamine dose. However, the presence of such a molecular response does not by itself establish its contribution to the acute behavioral phenotype.

The regional specificity of this response became particularly evident when mTOR signaling was considered across brain regions. mTOR is strongly implicated in ketamine-induced synaptic plasticity and antidepressant-like behavior, particularly through mechanisms involving protein synthesis and synaptogenesis [6,23,26]. In contrast to the striatum, neither ketamine dose restored the marked CUS-induced reduction in hippocampal pmTOR/mTOR, indicating that recovery of hippocampal mTOR signaling was not required for the acute antidepressant-like response observed at either dose. By comparison, 10 mg/kg restored relative mTOR activation in the striatum, where mTOR activation was positively associated with BDNF expression. Thus, mTOR responses further illustrate the regional divergence of ketamine-induced signaling and the broader recruitment of striatal plasticity-related pathways by the higher dose.

JNK signaling added another layer of complexity. JNK is a stress-responsive MAPK whose effects depend on cellular context, signal duration, and interaction with other signaling pathways [32,33]. In the present study, the molecular response of JNK to ketamine was strongly dependent on both dose and brain region. The more pronounced JNK response to the higher ketamine dose in the PFC and hippocampus, together with the absence of detectable changes in the striatum, suggests that JNK represents a stress-responsive component of ketamine signaling selectively recruited across stress-sensitive circuits rather than a uniform molecular correlate of antidepressant-like efficacy. This interpretation is also consistent with our previous study, in which sustained antidepressant-like effects after Ket/nBNI treatment were accompanied by inverse relationships between JNK and mTOR activation in the PFC and striatum [7].

BDNF regulation also followed a strongly region-dependent pattern. Both ketamine doses increased BDNF levels in the PFC, reduced them in the hippocampus, and restored the marked CUS-induced decrease in striatal BDNF, with the strongest effect observed after 10 mg/kg. These divergent responses are consistent with the region-specific nature of BDNF function in stress and depression, in which changes in BDNF expression and signaling can have different functional consequences depending on the brain region and circuit involved [34]. The positive correlations between BDNF and mTOR in the hippocampus, and between BDNF and both ERK and mTOR in the striatum, are consistent with evidence linking BDNF–TrkB signaling to downstream ERK and mTOR pathways after ketamine treatment [24,25,27]. Together, these findings indicate that ketamine engages distinct regional BDNF-associated signaling states rather than a uniform brain-wide neurotrophic response.

The reduction in hippocampal BDNF should also be interpreted in the context of the experimental model. Ketamine was administered 30 days after adolescent CUS, following a prolonged stress-free interval during which persistent molecular adaptations may have altered the subsequent response to treatment. Indeed, in our previous study using the same adolescent CUS paradigm, BDNF and associated signaling pathways showed marked region-specific regulation [7], indicating that the hippocampus does not necessarily follow a simple stress-induced decrease/ketamine-induced restoration pattern. Thus, ketamine in the present study acts on a molecular background shaped by long-term consequences of adolescent stress, which may account for differences from studies examining ketamine in other stress paradigms or experimental conditions. In addition, BDNF was assessed 48 h after treatment, whereas the canonical BDNF response to ketamine has primarily been associated with earlier signaling events. The temporal profile of hippocampal BDNF signaling is also relevant to the present findings. Kim et al. (2021) [35] showed that hippocampal BDNF protein increased rapidly after ketamine but returned to baseline by day 7, whereas MeCP2 phosphorylation emerged at the later time point and was linked to sustained antidepressant effects. Although our study did not assess MeCP2 signaling or molecular changes at one week, these findings support the view that early BDNF responses and later mechanisms maintaining antidepressant effects may be temporally distinct. Accordingly, the reduced hippocampal BDNF observed at 48 h in the present study does not exclude an earlier transient BDNF increase and may instead reflect a later, model-specific molecular state shaped by prior adolescent stress.

Exploratory behavior–molecular correlations further highlighted the striatum. Across all experimental groups, higher striatal JNK activation was associated with greater TST immobility, whereas higher striatal BDNF was associated with lower immobility. Because these analyses pooled CTRL, CUS, KET/6, and KET/10 animals, they primarily reflect covariation across experimental conditions rather than individual ketamine responsiveness.

Striatal BDNF was also negatively correlated with TST immobility within the KET/10 group, whereas no significant correlations were detected within KET/6. Together, these findings support a region- and pathway-specific relationship between molecular signaling and behavior, with striatal BDNF emerging as the clearest correlate in the present dataset. Given the small sample sizes and the 24 h interval between behavioral testing and molecular assessment, these correlations should be considered exploratory and hypothesis-generating.

**Figure 7:**
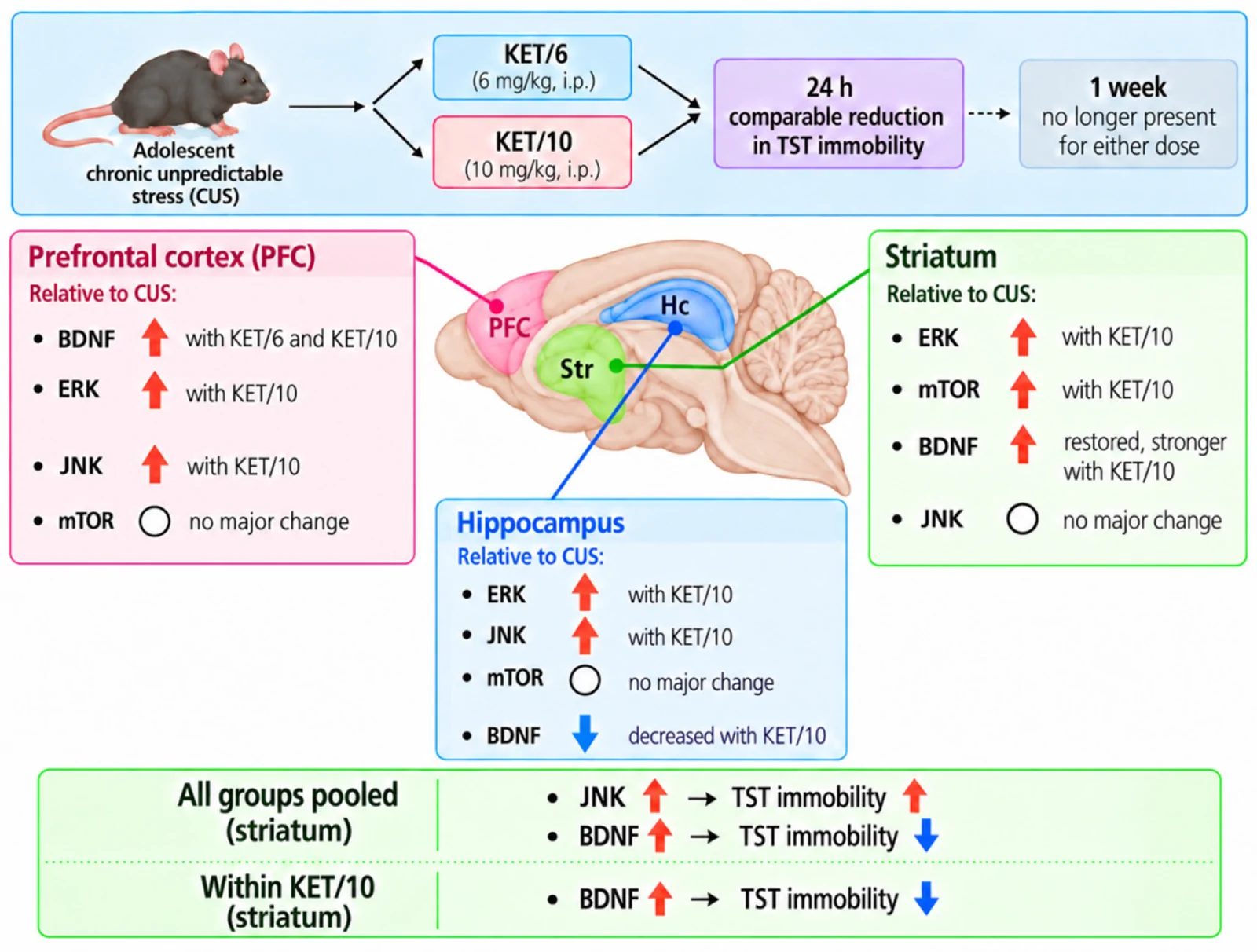
Summary of behavioral effects and region-specific molecular responses to ketamine. KET/6 and KET/10 produced comparable acute reductions in TST immobility despite distinct molecular responses across the PFC, hippocampus, and striatum. Exploratory correlation analyses identified striatal JNK and BDNF as molecular measures associated with TST immobility across experimental conditions, with the BDNF association also observed in the striatum within the KET/10 group.

Overall, the molecular effects of ketamine were strongly dose-, region-, and pathway-dependent. The broader signaling response induced by 10 mg/kg, despite behavioral efficacy comparable to 6 mg/kg, indicates that molecular pathway engagement cannot be interpreted solely by its magnitude. Instead, regional and temporal context appear critical for understanding the relationship between ketamine-induced signaling and antidepressant-like behavior (Figure 7).

### Limitations

Several limitations should be considered when interpreting these findings. First, molecular analyses were performed at a single 48 h time point after ketamine administration, corresponding to 24 h after behavioral testing. This time point captures molecular changes associated with the post-treatment period but does not define the full temporal profile of ketamine action. ERK, mTOR, JNK, and BDNF signaling are dynamic, and earlier or later measurements may reveal transient responses involved in initiating the behavioral effect or delayed adaptations related to its persistence. Future studies incorporating multiple post-treatment intervals would therefore help distinguish molecular events associated with response onset from those involved in sustained antidepressant-like effects.

An additional limitation is that the one-week behavioral cohort underwent OFT and TST testing at both 24 h and one week after ketamine administration. Previous exposure to these behavioral paradigms may influence subsequent performance through habituation, learning, or test-related stress. Although all groups within this cohort were subjected to the same testing schedule, this factor should be considered when interpreting the one-week results. Accordingly, the one-week data are interpreted primarily as an assessment of whether the ketamine-induced behavioral effect was sustained over time, rather than as a direct comparison with the independently tested 24 h cohort.

Molecular measurements were performed in whole-region homogenates from the PFC, hippocampus, and striatum. This approach enabled direct comparison of the same signaling pathways across regions, but it does not resolve subregional or cell-type-specific effects. The observed changes may therefore reflect combined responses of distinct neuronal and non-neuronal populations, and studies with greater anatomical and cellular resolution will be required to identify the specific circuit and cellular components involved.

The study was conducted exclusively in male mice, which limits the generalization across sexes. Given known sex-dependent differences in stress responsivity and antidepressant treatment effects, it will be important to determine whether the dose- and region-specific behavioral and molecular responses observed here are similarly expressed in females.

Finally, the present study identifies associations between ketamine dose, regional signaling, and behavior but does not establish causal relationships between individual pathways and antidepressant-like behavior. Direct pharmacological or cell-specific manipulation of ERK, mTOR, JNK, and BDNF signaling will be required to define their functional contribution. In addition, behavioral assessment was limited to the TST and OFT; inclusion of complementary domains, such as anhedonia, motivation, or social behavior, would provide a broader evaluation of ketamine’s behavioral effects.

## 4. Materials and Methods

### 4.1. Animals

All experiments were performed using adolescent male C57BL/6J mice originally obtained from The Jackson Laboratory and subsequently bred in our animal facility. Mice were housed three to four per cage under controlled environmental conditions (20 ± 2 °C) with a 12 h light/dark cycle, and had free access to standard laboratory chow and water. All procedures were approved by the Ethical Committee of the VINCA Institute of Nuclear Sciences (approval no. 323-07-03268/023-05) and were carried out in compliance with the European Communities Council Directive 2010/63/EU for the protection of animals used for scientific purposes.

Animals were randomly assigned to experimental groups and housed in stable cage groups throughout the study; animals were not regrouped following CUS. Following stress exposure, the CUS cohort was further divided into three treatment subgroups, as detailed in Section 4.2.

### 4.2. Chronic Unpredictable Stress (CUS)

Chronic stress represents a critical environmental factor in the pathophysiology of mood disorders, including depression and anxiety, as it induces long-lasting behavioral and neurobiological changes. To model these alterations experimentally, we utilized the CUS paradigm, a widely accepted and validated procedure for eliciting depression-like phenotypes in rodents [7,10,11,12].

Adolescent male mice were subjected to a 12-day CUS protocol (Table 2), beginning on postnatal day 28 (P28). This duration was chosen based on previous studies demonstrating that, during adolescence—a developmental window marked by increased vulnerability to stress—even relatively brief stress exposure is sufficient to produce stable depression-like behavioral outcomes [7,10,11,12]. Throughout the protocol, animals were exposed to three stressors per day during the light phase (morning, afternoon, and evening) in dedicated procedure rooms. To minimize habituation and maintain stress responsivity, different physical and social stressors were applied in a variable sequence each day. Between individual stress sessions, mice were returned to their home cages. Upon completion of the CUS procedure, animals underwent a 30-day recovery period (P40–P70), allowing transition into adulthood prior to subsequent behavioral assessments and pharmacological interventions.

Body weight was monitored throughout the experimental period as previously described and reported [7]. Briefly, animals were weighed at defined time points during the CUS procedure and recovery phase to assess stress-induced alterations in somatic growth. Because these data were previously reported for the same CUS cohort, they are provided here as supportive physiological information in Supplementary Table S1 and Supplementary Figure S3.

Animals that were not subjected to the CUS protocol served as non-stressed controls. Following the 30-day recovery period, a total of 80 mice were allocated to two independent experimental cohorts (n = 40/cohort), each comprising CTRL, CUS, KET/6, and KET/10 groups (n = 10/group). Animals were randomly assigned to experimental groups and housed in stable cage groups throughout the study; animals were not regrouped following CUS. The individual mouse was considered the experimental unit. Within each cohort, control animals (CTRL, n = 10) received a single intraperitoneal (i.p.) injection of vehicle (0.9% NaCl), administered in a body weight-adjusted volume; CUS-exposed mice were randomly assigned to three experimental groups (n = 10 per group) and treated on postnatal day 70 (P70). The first group (CUS) received an i.p. injection of vehicle (0.9% NaCl). The second group (KET/6) was administered ketamine (Ketamidor, Richter Pharma AG, Wels, Austria) at a dose of 6 mg/kg, dissolved in saline. The third group (KET/10) received ketamine at 10 mg/kg (i.p.). The 6 and 10 mg/kg doses were selected as two closely spaced doses within the range commonly used to elicit antidepressant-like effects in mice [4]. This design allowed us to ask whether comparable behavioral efficacy within the antidepressant-effective dose range is accompanied by comparable molecular signaling.

To assess the impact of chronic stress and ketamine treatment on locomotor activity and depression-like behavior, animals were assigned to two independent experimental cohorts. One cohort underwent behavioral testing 24 h after drug administration using the open field test (OFT) and the tail suspension test (TST). Upon completion of behavioral assessment, animals were left undisturbed for an additional 24 h and subsequently sacrificed, resulting in tissue collection 48 h after drug administration. Animals were euthanized by decapitation at the designated time points, and brains were rapidly collected for molecular analyses. The prefrontal cortex, hippocampus, and striatum were rapidly dissected and stored at −80 °C until further molecular analyses. The second cohort underwent the same behavioral testing 24 h after drug administration and was then maintained until one week after treatment, when the OFT and TST were repeated. The 24-h behavioral data from the second cohort and the corresponding molecular analyses are not included in the present study; only the one-week behavioral data from this cohort are presented. Thus, the one-week behavioral measurements were obtained from animals that had previously undergone the same behavioral tests at 24 h.

### 4.3. Behavioral Assessment

Open field test (OFT)

Locomotor activity and anxiety-related behavior were evaluated using the open field test. The apparatus consisted of a square white arena (520 × 520 mm). Prior to testing, animals were allowed to acclimatize to the testing room for 30 min to minimize the effects of handling and environmental novelty. Each animal was individually placed in the center of the arena and allowed to explore freely for 10 min under an illumination of approximately 80 lux in a quiet testing room. Movements were recorded by an automated video-tracking system and analyzed using TSE VideoMot 2 software (TSE Systems, Bad Homburg, Germany).

The primary parameters analyzed were total path length, reflecting general locomotor activity, and path length in the central zone of the arena, used as an indicator of anxiety-like behavior [21]. Behavioral testing was performed 24 h following drug administration. Between animals, the arena was thoroughly cleaned to minimize olfactory cues and disinfected with 70% ethanol, followed by drying before the next animal was tested. After completion of the OFT, animals were returned to their home cages prior to further procedures. Behavioral testing and scoring were performed with the evaluator blinded to experimental group assignment.

Tail suspension test (TST)

Depression-like behavior was assessed using the tail suspension test. Mice were suspended by the tail using adhesive tape affixed approximately 1–2 cm from the tip, ensuring that the body hung freely without contacting surrounding surfaces. To eliminate visual and physical interaction, animals were tested individually in separate compartments. Each session lasted 6 min and was recorded for subsequent analysis. Immobility time, defined as the absence of active escape-directed movements, was quantified. Scoring was conducted offline by observers blinded to treatment allocation. Following each test, the apparatus was cleaned between animals with 70% ethanol to minimize residual olfactory cues [22].

### 4.4. Western Blot Analysis

Frozen brain samples were weighed and homogenized (1:10, *w*/*v*) in ice-cold lysis buffer containing 50 mM Tris-HCl (pH 7.5), 150 mM NaCl, 1% NP-40, 0.1% SDS, 10 mM EDTA (pH 8), 10 mM EGTA (pH 7.2), 0.5% Triton X-100, and phosphatase inhibitors (20 mM β-glycerophosphate, 5 mM Na_4_P_2_O_7_•10H_2_O, 2 mM Na_3_VO_4_, 25 mM NaF). Homogenization was performed using 30 strokes with a Potter–Elvehjem Teflon-glass homogenizer. Homogenates were centrifuged at 14,000 rpm for 30 min at 4 °C, and the resulting supernatants were collected, aliquoted, and stored at −80 °C until analysis.

Protein concentrations were determined using the method of Markwell et al. [36]. Samples were then mixed with Laemmli denaturing buffer and heated at 100 °C for 5 min [37]. Forty micrograms of protein per sample were separated by SDS-PAGE on 7.5%, 10%, or 12% polyacrylamide gels and subsequently transferred to PVDF membranes (Immobilon-P, Millipore, Billerica, MA, USA) using a Transblot system (Bio-Rad Laboratories, Hercules, CA, USA). Membranes were blocked for 1 h at room temperature with 5% non-fat milk in PBS, followed by overnight incubation at 4 °C with primary antibodies diluted in 2.5% milk in PBS-Tween (PBS-T), as detailed in Table 3. After three washes in PBS-T, membranes were incubated with HRP-conjugated secondary antibodies (rabbit anti-mouse IgG-HRP or goat anti-rabbit IgG-HRP; Abcam, Cambridge, UK) for 1 h at room temperature. Immunoreactive bands were detected using enhanced chemiluminescence (SuperSignal Pico or Femto, Thermo Scientific, Waltham, MA, USA) and exposed to X-ray film (Amersham Hyperfilm ECL, Cytiva, Marlborough, MA, USA).

Band intensities were quantified using ImageJ analysis software v1.54 p (National Institutes of Health, Bethesda, MD, USA) and normalized to total protein levels visualized by Ponceau S staining within the 40–70 kDa molecular-weight range. Phosphorylated and corresponding total protein forms were assessed on the same membrane, allowing calculation of phospho-to-total protein ratios. To allow comparison between gels, an internal reference standard (IRS) was loaded on every gel. The IRS consisted of pooled homogenate from control animals, and the same IRS preparation was used throughout the experiments. Relative protein expression for each sample was calculated as a percentage of the IRS, ensuring consistency and enabling quantitative comparison across experiments.

### 4.5. Statistical Analysis

Data were analyzed using GraphPad Prism software (v.6, San Diego, CA, USA). Data distributions were assessed for normality using the Shapiro–Wilk test and for homogeneity of variance using the Brown–Forsythe test prior to statistical analysis. Sample sizes were determined a priori based on previous experimental evidence and pilot experiments using the same CUS paradigm. Behavioral analyses were performed in up to n = 10 animals per group. For molecular analyses, a subset of n = 7 animals per group was selected from the same behavioral cohort. Final sample sizes for individual molecular endpoints were further reduced in some cases because of technical/sample loss or exclusion according to the predefined median ±2 median absolute deviations (MAD) criterion within each group (Supplementary Table S2). For comparisons among multiple groups, one-way analysis of variance (ANOVA) was performed, followed by Tukey’s multiple comparisons test. Pearson correlation coefficients (r) were calculated to assess associations between BDNF expression and kinase activation within each brain region and between TST immobility and ERK, mTOR, JNK, and BDNF measures. Behavior–molecular correlations were examined across the complete experimental population (CTRL, CUS, KET/6, and KET/10) and separately within the KET/6 and KET/10 groups. Correlations based on fewer than five paired observations were reported for completeness but were not interpreted. Correlation analyses were considered exploratory. All data are expressed as mean ± standard error of the mean (SEM), and *p* < 0.05 was considered statistically significant.

## 5. Conclusions

The present study demonstrates that comparable acute antidepressant-like effects of two subanesthetic ketamine doses can be accompanied by different region-specific molecular responses. In particular, the higher dose recruited broader ERK-, mTOR-, JNK-, and BDNF-related signaling without producing a greater reduction in TST immobility, indicating that the extent of molecular engagement is not directly proportional to acute behavioral efficacy. The striatum emerged as a particularly responsive region, with dose-dependent restoration of BDNF and distinct signaling changes that were also associated with TST performance. These findings emphasize that ketamine dose–response relationships should be interpreted in a region-, pathway-, and time-dependent context rather than solely on the basis of acute behavioral effects. Future studies incorporating additional behavioral domains, both sexes, and multiple molecular time points will be important for determining which signaling responses contribute to the persistence of antidepressant-like effects and which reflect broader pharmacological engagement.

## Figures and Tables

**Table 1:** Summary of ketamine-induced molecular changes across brain regions.

Region	Marker	KET/6 vs. CUS	KET/10 vs. CUS	KET/10 vs. KET/6
PFC	ERK ratio	ns	↑	↑
PFC	mTOR ratio	ns	ns	ns
PFC	JNK ratio	ns	↑	↑
PFC	BDNF	↑	↑	ns
Hippocampus	ERK ratio	ns	↑	↑
Hippocampus	mTOR ratio	ns	ns	ns
Hippocampus	JNK ratio	ns	↑	↑
Hippocampus	BDNF	ns	↓	ns
Striatum	ERK ratio	ns	↑	↑
Striatum	mTOR ratio	ns	↑	↑
Striatum	JNK ratio	ns	ns	ns
Striatum	BDNF	↑	↑	↑

Note: Arrows indicate statistically significant increases or decreases. “ns” indicates no significant difference. ERK, mTOR, and JNK refer to phospho-to-total protein ratios. BDNF refers to total BDNF protein expression. Direct KET/10 vs. KET/6 comparisons included are statistically significant.

**Table 2:** Daily schedule of stressors applied during the 12-day chronic unpredictable stress (CUS) procedure.

Day	Morning (09:00)	Afternoon (14:00)	Evening (18:00)
1.	—	—	Prolonged light exposure
2.	Cold exposure (4 °C, 60 min)	Dark phase induction/cage tilting (3 h)	Food deprivation
3.	Cage tilting (45°, 3 h)	Cold stress with cage shaking (60 min)	Intermittent strobe light
4.	Predator odor exposure (3 h)	Forced swim (18 °C, 10 min)	Damp bedding overnight
5.	Restraint stress (60 min)	Cold exposure (60 min)	Cage tilting
6.	Dark phase induction (3 h)	Cold stress with shaking (60 min)	Prolonged light exposure
7.	Cold exposure (60 min)	Predator odor (3 h)	Intermittent strobe light
8.	Restraint (60 min)	Dark phase induction/cage tilting (3 h)	Damp bedding
9.	Forced swim (18 °C, 10 min)	Predator odor (3 h)	Food deprivation
10.	Restraint (60 min)	Cold exposure (60 min)	Intermittent strobe light
11.	Social stress (novel cage mate, 3 h)	Restraint (60 min)	Light exposure without bedding
12.	Forced swim (10 min)	Cold stress with shaking (60 min)	Damp bedding

**Table 3:** Primary antibodies used for Western blotting in the present study.

Protein	Dilution for WB	Company	Cat. No.
p mTOR (Ser 2448)	1:500	Santa Cruz Biotechnology, Dallas, TX, USA	sc-101738
mTOR	1:500	Santa Cruz Biotechnology, Dallas, TX, USA	sc-517464
pErk 1/2 (Thr202/Tyr 204)	1:1000	Cell Signaling, Danvers, MA, USA	#9101
Erk 1/2	1:1000	Cell Signaling, Danvers, MA, USA	#9102
pJNK (Thr 183/Tyr 185)	1:500	Santa Cruz Biotechnology, Dallas, TX, USA	sc-6254
JNK	1:2000	Santa Cruz Biotechnology, Dallas, TX, USA	sc-571
BDNF	1:2000	Abcam, Cambridge, UK	ab108319

Abbreviations—pmTOR: phosphorylated mammalian target of rapamycin; mTOR: mammalian target of rapamycin; pErk1/2: phosphorylated extracellular signal-regulated kinase 1/2; Erk1/2: extracellular signal-regulated kinase 1/2; pJNK: phosphorylated c-Jun N-terminal kinase; JNK: c-Jun N-terminal kinase; BDNF: brain-derived neurotrophic factor.

## Data Availability

The original contributions presented in this study are included in the article. Further inquiries can be directed to the corresponding author.

## Supplementary Materials

The supporting information can be downloaded at https://www.mdpi.com/article/10.3390/ijms27209126/s1.

## Abbreviations

The following abbreviations are used in this manuscript:

ANOVA	Analysis of variance
BDNF	Brain-derived neurotrophic factor
BTs	Behavioral tests
CTRL	Non-stressed control group
CUS	Chronic unpredictable stress
ERK	Extracellular signal-regulated kinase
HIPP	Hippocampus
JNK	c-Jun N-terminal kinase
KET	Ketamine
KET/6	Chronic unpredictable stress followed by ketamine treatment at 6 mg/kg
KET/10	Chronic unpredictable stress followed by ketamine treatment at 10 mg/kg
MDD	Major depressive disorder
mTOR	Mechanistic target of rapamycin
NMDA	N-Methyl-D-aspartate
NMDAR	N-Methyl-D-aspartate receptor
ns	Not significant
OFT	Open field test
pERK	Phosphorylated extracellular signal-regulated kinase
PFC	Prefrontal cortex
p-JNK	Phosphorylated c-Jun N-terminal kinase
p-mTOR	Phosphorylated mechanistic target of rapamycin
SEM	Standard error of the mean
STR	Striatum
tERK	Total extracellular signal-regulated kinase
tJNK	Total c-Jun N-terminal kinase
tmTOR	Total mechanistic target of rapamycin
TST	Tail suspension test
1W	One week
