# Supplementary material for: Comparable Acute Antidepressant-like Effects of Two Subanesthetic Ketamine Doses Are Associated with Divergent Region-Specific Molecular Responses After Adolescent Chronic Stress
Source: Int J Mol Sci. 2026 Oct 15;27(20):9126. doi: 10.3390/ijms27209126 (PMC13704947; doi:10.3390/ijms27209126)
Supplement: Supplementary file 1 [file ijms-27-09126-s001.zip › ijms-4602312-Supplementary data.pdf]

Supplementary correlation analysis

Pearson correlations were calculated between individual TST immobility values and ERK, mTOR, JNK, and BDNF measurements obtained from corresponding animals in the PFC, hippocampus, and striatum. Two complementary analyses were performed. First, CTRL, CUS, KET/6, and KET/10 animals were pooled to identify molecular measures associated with the overall behavioral phenotype across experimental conditions. Second, correlations were calculated separately within the KET/6 and KET/10 groups to assess associations between molecular signaling and behavioral outcome within each ketamine dose. The complete correlation matrices are presented in Supplementary Figures S1 and S2. Correlations based on fewer than five paired observations are shown for completeness but were not interpreted because of the instability of correlation estimates at very small sample sizes. All correlation analyses were considered exploratory.

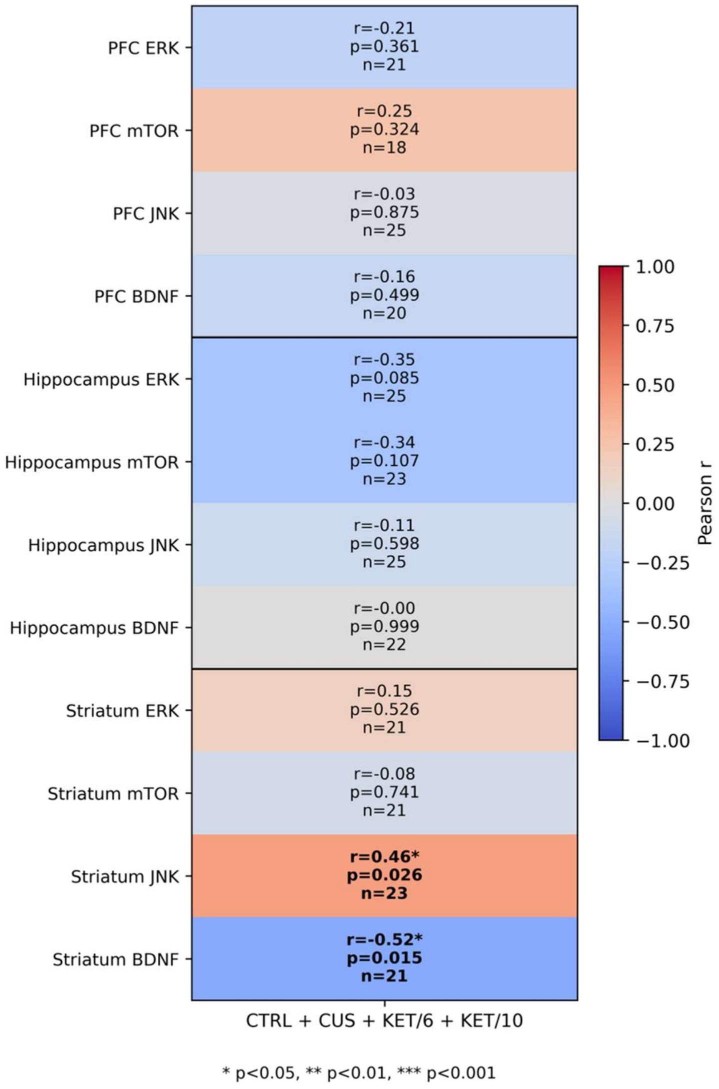

Supplementary Figure S1. Complete correlation matrix between TST immobility and regional molecular signaling markers across the complete experimental population. Pearson correlations were calculated between

individual TST immobility values and ERK, mTOR, JNK, and BDNF measurements obtained from corresponding animals in the PFC, hippocampus, and striatum after pooling CTRL, CUS, KET/6, and KET/10 groups. Heatmap colors represent Pearson correlation coefficients (r), with positive correlations shown in red and negative correlations in blue. Each cell reports Pearson r, p value, and the number of paired observations (n). Significant associations were detected for striatal p-JNK/JNK (r = 0.46, p = 0.026, n = 23) and striatal BDNF (r = −0.52, p = 0.015, n = 21). *p < 0.05, **p < 0.01, ***p < 0.001.

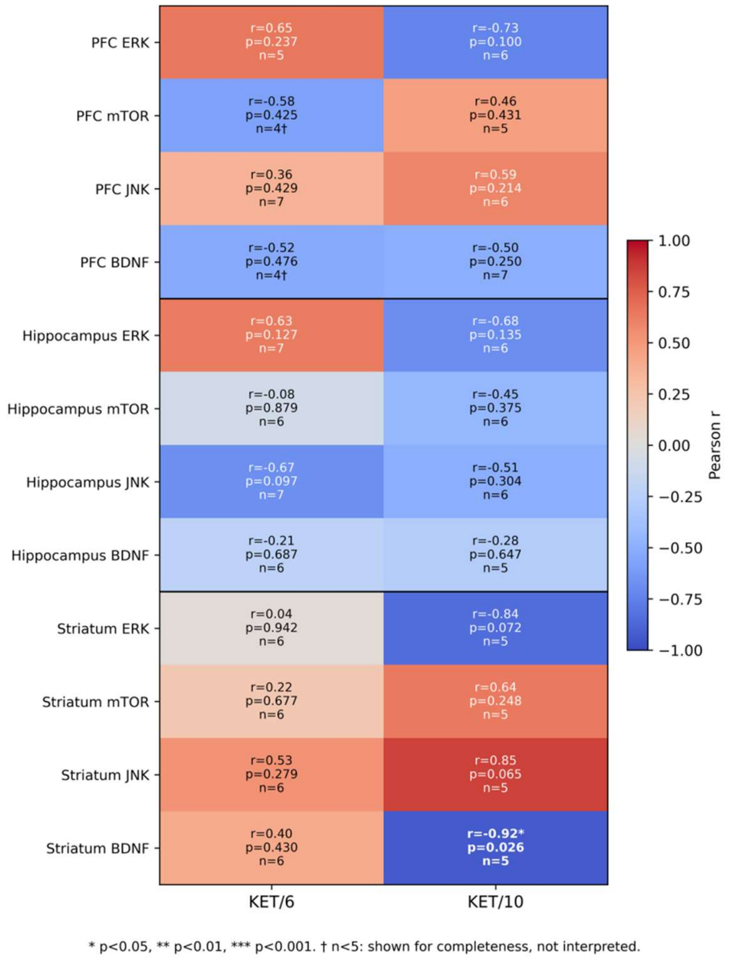

Supplementary Figure S2. Complete dose-specific correlation matrices between TST immobility and regional molecular signaling markers in ketamine-treated animals. Pearson correlations were calculated between individual TST immobility values and ERK, mTOR, JNK, and BDNF measurements obtained from corresponding animals separately within the KET/6 and KET/10 groups. Heatmap colors represent Pearson correlation coefficients (r), with positive correlations shown in red and negative correlations in blue. Each cell reports Pearson r, p value, and the number of paired observations (n). No significant correlations were detected within the KET/6 group. Within the KET/10 group, striatal BDNF expression was negatively correlated with TST immobility (r = −0.92, p = 0.026, n = 5). *p < 0.05, **p < 0.01, ***p < 0.001. Correlations based on fewer than five paired observations are marked with † and are shown for completeness but were not interpreted.

<u>Body-weight changes following CUS</u>

Body-weight gain was monitored during the adolescent CUS period (P28–P40) and during the subsequent stress-free period (P40–P70) (Suppl. Figure S3, Supplementary Table S1). As previously reported for this cohort (Zivanovic et al., 2026), CUS-exposed mice showed markedly reduced body-weight gain during the stress period compared with non-stressed controls. During the subsequent stress-free interval, the pattern reversed, with CUS-exposed mice showing greater body-weight gain than controls, consistent with compensatory weight recovery after cessation of stress. These data are provided as supportive physiological information and were not used as a primary measure of stress susceptibility or antidepressant response.

Supplementary Table S1. Individual body-weight changes during adolescent CUS and the subsequent stress-free period. Individual changes in body weight are shown for control and CUS-exposed mice during the stress period (P28–P40) and the subsequent stress-free period (P40–P70). Values are expressed in grams. These data were previously reported for the same CUS cohort in Zivanovic et al. (2026).

STRESS (P28-P40) (g)		REST (P40-P70) (g)	
CONTROL	CUS	CONTROL	CUS
7.26	5.54	7.24	6.28
7.81	4.64	5.90	8.22
8.26	4.70	5.97	7.49
8.23	5.64	6.46	9.84
8.02	3.57	7.24	7.24
8.37	5.65	5.55	6.72
9.78	5.26	6.42	10.20
8.24	5.66	5.90	8.07
8.74	3.65	6.46	9.02
9.81	5.17	5.97	7.61
	5.68		8.18
	4.68		8.33
	3.80		6.78
	3.87		7.74
	4.74		9.17
	6.64		7.42
	4.58		9.05
	3.90		7.35
	4.11		8.53
	3.65		9.28
	4.66		7.79
	4.65		7.53
	5.25		7.09
	5.67		10.38
	4.99		9.43
	5.01		6.62
	5.78		8.29
	3.72		8.55
	4.52		11.14
	5.77		8.04

Body weight changes following chronic unpredictable stress (CUS)

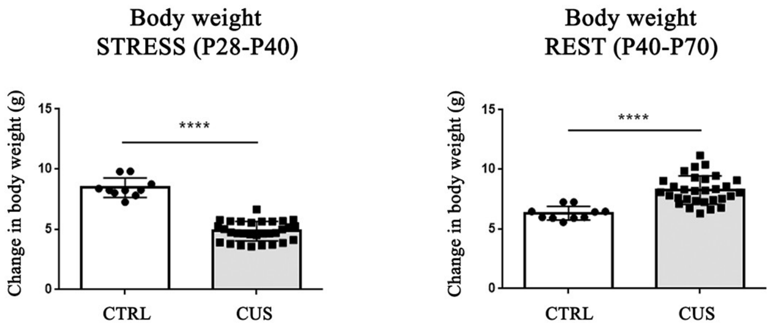

Supplementary Figure S3. Body-weight gain during adolescent chronic unpredictable stress and the subsequent stress-free period. Body-weight gain was calculated during the CUS period (P28–P40) and during the subsequent stress-free recovery period (P40–P70). CUS-exposed mice showed reduced body-weight gain during the stress period compared with controls, whereas during the recovery period they showed greater weight gain than non-stressed controls. Data are presented as mean ± SEM with individual animals shown. CTRL, non-stressed control; CUS, chronic unpredictable stress. ****$p < 0.0001$. These data were previously reported for the same CUS cohort in Zivanovic et al. (2026).

Outlier exclusion

Outliers were identified using the predefined median ±2 MAD criterion. Only analyses in which at least one MAD-defined outlier was excluded are shown. For molecular analyses, technical/sample losses affecting the same analysis are also indicated. Behavioral analyses started with n=10 animals per group, whereas molecular analyses were performed on a subset of n=7 animals per group.

Supplementary Table S2. Sample exclusions and final sample sizes for behavioral and molecular analyses.

Analysis	Measure	Group	Initial n	Technical/sample loss	MAD exclusion	MAD value(s)	Final n
Behavioral	TST immobility	KET/6	10	0	1	7	9
Behavioral	TST immobility	KET/10	10	0	3	33; 252; 260	7
Molecular	PFC ERK	CUS	7	1	1	1.95093	5
Molecular	PFC BDNF	CTRL	7	2	1	24.2338	4
Molecular	PFC BDNF	CUS	7	1	1	76.2409	5
Molecular	PFC BDNF	KET/6	7	2	1	29.6098	4
Molecular	HIPP JNK	CUS	7	0	1	5.22526	6
Molecular	STR mTOR	CUS	7	1	1	1.55861	5

Molecular	STR JNK	KET/6	7	0	1	1.84514	6
Molecular	STR BDNF	CTRL	7	1	1	39.0665	5
Molecular	STR BDNF	CUS	7	1	1	60.0813	5
